# Synthesis of Small Ce^3+^-Er^3+^-Yb^3+^ Tri-Doped BaLuF_5_ Active-Core-Active-Shell-Active-Shell Nanoparticles with Strong Down Conversion Luminescence at 1.5 μm

**DOI:** 10.3390/nano8080615

**Published:** 2018-08-14

**Authors:** Yongling Zhang, Yudi Shi, Zhengkun Qin, Mingxing Song, Weiping Qin

**Affiliations:** 1 College of Information &Technology, Jilin Normal University, Siping 136000, China; shiyudijlu@163.com; 2 State Key Laboratory on Integrated Optoelectronics, College of Electronic Science & Engineering, Jilin University, Changchun 130012, China; wpqin@jlu.edu.cn

**Keywords:** BaLuF_5_, nanoparticles, active-shell, core-shell, down conversion luminescence, 1.5 μm

## Abstract

Small fluoride nanoparticles (NPs) with strong down-conversion (DC) luminescence at 1.5 μm are quite desirable for optical fiber communication systems. Nevertheless, a problem exists regarding how to synthesize small fluoride NPs with strong DC emission at 1.5 μm. Herein, we propose an approach to improve 1.5 μm emission of BaLuF_5_:Yb^3+^,Er^3+^ NPs by way of combining doping Ce^3+^ ions and coating multiple BaLuF_5_: Yb^3+^ active-shells. We prepared the BaLuF_5_:18%Yb^3+^,2%Er^3+^,2%Ce^3+^ NPs through a high-boiling solvent method. The effect of Ce^3+^ concentration on the DC luminescence was systematically investigated in the BaLuF_5_:Yb^3+^,Er^3+^ NPs. Under a 980 nm laser excitation, the intensities of 1.53 μm emission of BaLuF_5_:18%Yb^3+^,2%Er^3+^,2%Ce^3+^ NPs was enhanced by 2.6 times comparing to that of BaLuF_5_:18%Yb^3+^,2%Er^3+^ NPs since the energy transfer between Er^3+^ and Ce^3+^ ions: Er^3+^:^4^I_11/2_ (Er^3+^) + ^2^F_5/2_ (Ce^3+^) → ^4^I_13/2_ (Er^3+^) + ^2^F_7/2_ (Ce^3+^). Then, we synthesized BaLuF_5_:18%Yb^3+^,2%Er^3+^,2%Ce^3+^@BaLuF_5_:5%Yb^3+^@BaLuF_5_:5%Yb^3+^ core-active-shell-active-shell NPs via a layer-by-layer strategy. After coating two BaLuF_5_:Yb^3+^ active-shell around BaLuF_5_:Yb^3+^,Er^3+^,Ce^3+^ NPs, the intensities of the 1.53 μm emission was enhanced by 44 times compared to that of BaLuF_5_:Yb^3+^,Er^3+^ core NPs, since the active-shells could be used to not only suppress surface quenching but also to transfer the pump light to the core region efficiently through Yb^3+^ ions inside the active-shells.

## 1. Introduction

Recently, trivalent rare-earth (RE^3+^) ions doped fluoride nanoparticles (NPs) have been applied widely in many fields of high technology, such as bioimaging, drug delivery, photodynamic therapy, solar cells [1,2,3,4,5,6,7,8,9,10,11,12], etc. In particular, Er^3+^-doped fluoride NPs have been applied in waveguide amplifiers [13,14,15,16] since intra-4f-shell transitions of Er^3+^ ions not only cause visible light emissions but also send an emission at 1.5 μm (the ^4^I_13/2_ → ^4^I_15/2_ transition of Er^3+^ ions), which is located in low loss windows of optical communication networks. In order to get high-gain Er^3+^-doped waveguide amplifiers, Er^3+^-doped fluoride NPs should not only have a strong down-conversion luminescence at 1.5 μm, but also have a small size. So far, various strategies have been developed to improve luminescence intensity of Er^3+^-doped fluoride NPs at 1.5 μm. One is to increase the nonradiative decay rate that the high energy levels of Er^3+^ ions relax nonradiatively to the ^4^I_13/2_ level [17,18,19]. Zhai et al. synthesized NaYF_4_:Er^3+^,Yb^3+^,Ce^3+^ NPs, and found the 1.53 μm emission band of Er^3+^ ions in the NPs was enhanced by 6 times after co-doping Ce^3+^ ions owing to the efficient energy transfer between Ce^3+^ and Er^3+^:^4^I_11/2_ (Er^3+^) + ^2^F_5/2_ (Ce^3+^) → ^4^I_13/2_ (Er^3+^) + ^2^F_7/2_ (Ce^3+^) [20]. The other strategy is to decrease the defects on the surface of NPs through growing an inert shell (the shell and the core NPs have similar lattice constants) around the core NPs [21,22,23,24,25]. Bo et al. reported that the intensity of the 1540 nm emission of LaF_3_:Yb^3+^,Er^3+^ core NPs was enhanced after coating a LaF_3_ inert shell since the coating inert shell method can suppress the surface quenching effect. [26]. The last strategy is to increase the rate of the pump light through coating an active shell (e.g., the shell containing Yb^3+^ icons) on the core NPs [27,28,29,30,31]. Zhai et al. reported a method to improve the intensity BaYF_5_:Yb^3+^,Er^3+^ NPs at 1.53 μm through doping Yb^3+^ ions into the BaYF_5_ shell. BaYF_5_:Yb^3+^,Er^3+^@BaYF_5_:Yb^3+^ inert-core-active-shell NPs were obtained and the intensity of the 1.53 μm was enhanced when compared to the BaYF_5_:Yb^3+^,Er^3+^ core NPs [32]. Despite, recent progress in this field, it is necessary to explore new approaches to achieve small NPs with strong down-conversion luminescence at 1.5 μm for applications regarding near infrared optical communication networks.

In this paper, we choose BaLuF_5_ as the matrix since its phase is a single crystalline phase [33]. We prepared BaLuF_5_:Yb^3+^,Er^3+^,Ce^3+^ NPs by a high-boiling solvent method and studied the effect of the Ce^3+^ concentration on the up-conversion (UC) emission and down-conversion (DC) emission (at 1.5 μm) of BaLuF_5_:Yb^3+^,Er^3+^ NPs. We synthesized BaLuF_5_:Yb^3+^,Er^3+^,Ce^3+^@BaLuF_5_:Yb^3+^ core-shell NPs via growing a BaLuF_5_:Yb^3+^ shell and investigated the effect of the Yb^3+^ concentration of the shell on the 1.5 μm emission of the BaLuF_5_ core-shell NPs. Finally, we compounded multi-layer BaLuF_5_ core-shell NPs via a layer-by-layer strategy, and obtained BaLuF_5_:Yb^3+^,Er^3+^,Ce^3+^@BaLuF_5_:Yb^3+^@BaLuF_5_:Yb^3+^ core-active-shell active-shell NPs with a strong down-conversion luminescence at 1.5 μm.

## 2. Materials and Methods

All chemicals were used directly without further purification. Lu(NO_3_)_3_·6H_2_O, Yb(NO_3_)_3_·6H_2_O, Er(NO_3_)_3_·6H_2_O, and Ce(NO_3_)_3_·6H_2_O were purchased from Sigma-Aldrich Chemicals (Shanghai, China). Oleic acid (OA), 1-octadecene (ODE) and Barium stearate were obtained by Alfa Aesar Company (Shanghai, China). NaOH, NH_4_F and stearic acid (C_17_H_35_COOH) were obtained from China National Pharmaceutical Group Corporation (Beijing, China).

### 2.1. Preparation of BaLuF_5_:Yb^3+^,Er^3+^ NPs and BaLuF_5_:Yb^3+^,Er^3+^ Core-Shell NPs

Synthesis of rare-earth stearate: 10 mmol rare-earth nitrate (Lu(NO_3_)_3_·6H_2_O, Yb(NO_3_)_3_·6H_2_O, Er(NO_3_)_3_·6H_2_O, or Ce(NO_3_)_3_·6H_2_O) and 10 mmol stearic acid were dissolved in 120 mL ethanol, and the system was kept at 80 °C for 30 min. Then, a 20 mL NaOH solution (containing 1.2 g NaOH) was added dropwise into the system. The resulting mixture was refluxed at 80 °C for another 10 h. The reaction product was washed with water and ethanol [34].

Synthesis of BaLuF_5_ nanoparticles: 0.5 mmol barium stearates, 0.5 mmol pre-prepared rare-earth stearate (Re(C_17_H_35_COO)_3_), 15 mL ODE, and 15 mL OA were added to a four-neck round-bottom reaction vessel. After the reaction, the mixture was heated to 150 °C for 30 min under an argon (Ar) flow. A 10 mmol methanol solution containing 0.12g NH_4_F was added dropwise into the reaction mixture, and the reaction mixture was heated to 50 °C for 30 min. Then, the reaction mixture was rapidly heated to 300 °C for 1 h and cooled to room temperature (RT) under an Ar flow. The reaction product was washed with cyclohexane and ethanol [31]. The finally obtained nanoparticles were dispersed into cyclohexane. 

Synthesis of BaLuF_5_ core-shell nanoparticles: 0.5 mmol barium stearates, 0.5 mmol pre-prepared rare-earth stearate (Re(C_17_H_35_COO)_3_), 15 mL ODE, and 15 mL OA were added to a four-neck round-bottom reaction vessel. The reaction system was heated to 150 °C for 30 min under an Ar flow. After the reaction system was cooled down to 60 °C. The core nanoparticles cyclohexane solution was added into the reaction system with vigorous stirring. A 10 mmol methanol solution containing 0.12 g NH_4_F was added dropwise into the reaction system, and the system was heated to 50 °C for 30 min. Then the reaction mixture was rapidly heated to 300 °C for 1 h and cooled to RT under an Ar flow. The reaction product was washed with cyclohexane and ethanol.

Synthesis of BaLuF_5_ core-shell-shell nanoparticles: To coat the second layer of the shell, these as-synthesized core-shell NPs were used as seeds. The same coating process was repeated. BaLuF_5_ core-shell-shell nanoparticles were obtained.

### 2.2. Characterization

The X-ray powder diffraction (XRD, Rigaku, Japan) were collected by Model Rigaku Ru-200b with Cu K_α_ (40 kV, 40 Ma) irradiation (λ = 1.5406 Å). The scan range was set from 10° to 70°. The morphology of the particles was characterized by a JEM-2100F electron microscope (Tokyo, Japan) at 200 kV. The up-conversion spectra of the samples were recorded by a Hitachi F-4500 fluorescence spectrophotometer (Tokyo, Japan) at room temperature under the excitation of a 980 nm laser diode with a fixed power density of 70 W·cm^−2^ (1.0 nm for slit resolution and 700 V for PMT voltage). The DC spectra of the samples were collected by a SPEX 1000M spectrometer (HORIBA Group, Kyoto, Japan) at room temperature under the excitation of a 980 nm laser diode with a fixed power density of 70 W·cm^−2^ (2 mm for slit width). 

## 3. Results and Discussion

### 3.1. Crystal Structure and Morphology

**Figure 1:**
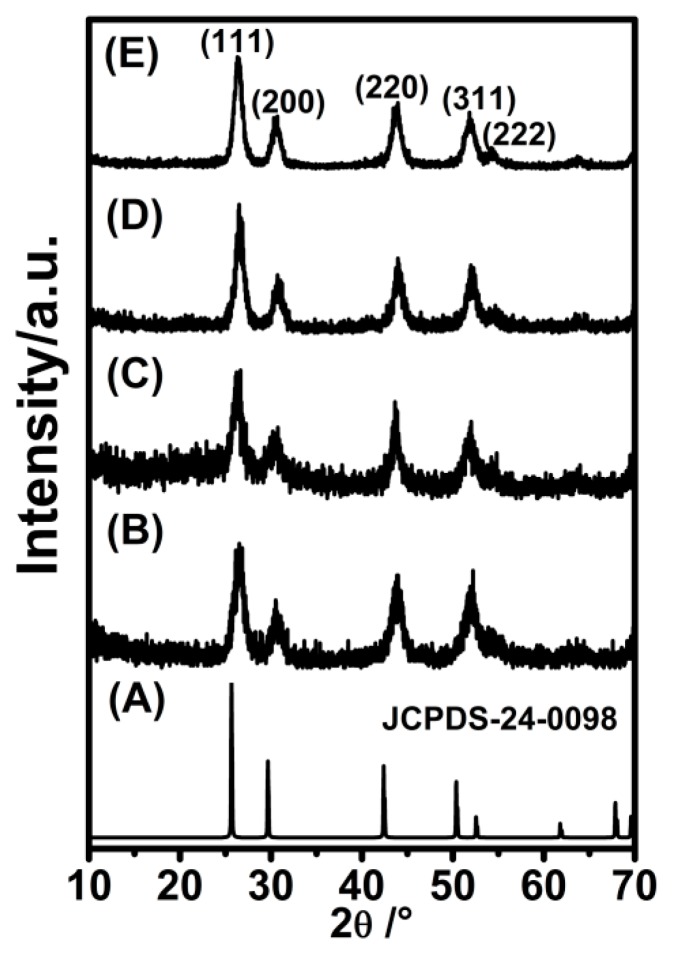
X-ray powder diffraction (XRD) patterns of (**A**) standard BaGdF_5_ NPs (the vertical bars denote the standard data for tetragonal structure of bulk BaGdF_5_ NPs (JCPDS-24-0098)); (**B**) BaLuF_5_:18%Yb^3+^,2%Er^3+^ core NPs; (**C**) BaLuF_5_:18%Yb^3+^,2%Er^3+^,2%Ce^3+^ core NPs; (**D**) BaLuF_5_:18%Yb^3+^,2%Er^3+^,2%Ce^3+^@BaLuF_5_:5%Yb^3+^ core-active-shell NPs; and (**E**) BaLuF_5_:18%Yb^3+^,2%Er^3+^,2%Ce^3+^@BaLuF_5_:5%Yb^3+^@BaLuF_5_:5%Yb^3+^ core-active-shell-active-shell NPs.

**Figure 2:**
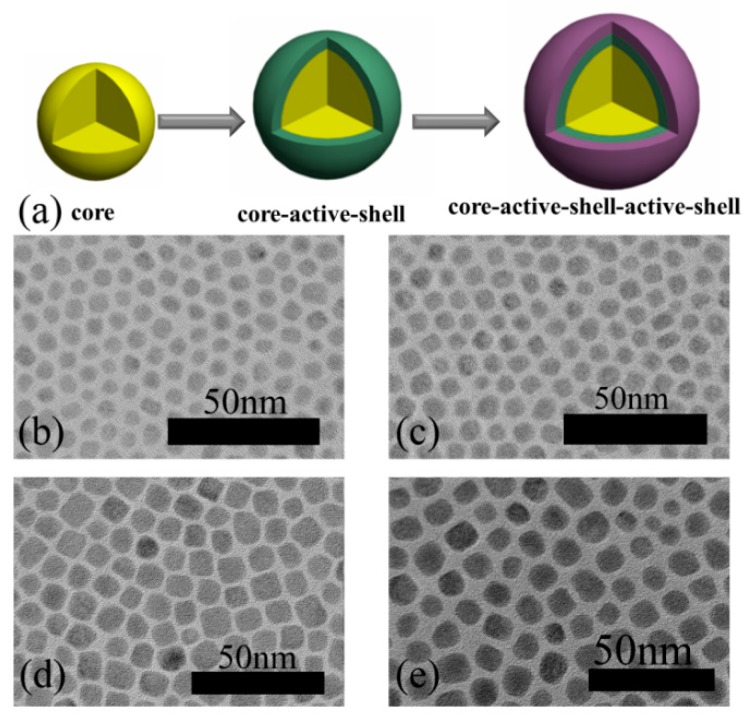
(**a**) Schematic illustration of BaLuF_5_ core NPs, BaLuF_5_ core-active-shell NPs and BaLuF_5_ core-active-shell-active-shell NPs. TEM images of (**b**) BaLuF_5_:18%Yb^3+^,2%Er^3+^ core NPs; (**c**) BaLuF_5_:18%Yb^3+^,2%Er^3+^,2%Ce^3+^ core NPs; (**d**) BaLuF_5_:18%Yb^3+^,2%Er^3+^,2%Ce^3+^@BaLuF_5_:5%Yb^3+^ core-active-shell NPs; and (**e**) BaLuF_5_:18%Yb^3+^,2%Er^3+^,2%Ce^3+^@BaLuF_5_:5%Yb^3+^@BaLuF_5_:5%Yb^3+^ core-active-shell-active-shell NPs.

The XRD patterns of the BaLuF_5_:18%Yb^3+^,2%Er^3+^ NPs, BaLuF_5_:18%Yb^3+^,2%Er^3+^,2%Ce^3+^ NPs, BaLuF_5_:18%Yb^3+^,2%Er^3+^,2%Ce^3+^@BaLuF_5_ core-inert-shell NPs, BaLuF_5_:18%Yb^3+^,2%Er^3+^,2%Ce^3+^@BaLuF_5_:5%Yb^3+^ core-active-shell NPs and BaLuF_5_:18%Yb^3+^,2%Er^3+^,2%Ce^3+^@BaLuF_5_:5%Yb^3+^@BaLuF_5_:5%Yb^3+^ core-active-shell active-shell NPs are shown in Figure 1. It shows that all the diffraction peaks of the samples were well-assigned to the tetragonal phase BaGdF_5_ (JCPDS No. 24-0098), which indicates that the samples are BaLuF_5_ nanoparticles. To characterize the morphology of the samples, we also measured the TEM images of the above samples, and the results are shown in Figure 2. From the TEM image (Figure 2), it is easily seen that the samples are round without agglomeration. The average sizes of BaLuF_5_:Yb^3+^,Er^3+^ core-only NPs and BaLuF_5_:Yb^3+^,Er^3+^,Ce^3+^ core-only NPs were both about 6 nm, and the above results show that the doping Ce^3+^ ions have not changed the size of BaLuF_5_ core-only NPs. The average size of the BaLuF_5_ core-active-shell NPs was about 8 nm after the epitaxial growth of a shell layer. The size of the BaLuF_5_ core-active-shell-active-shell NPs was further increased to about 10 nm after the growth of two shell layers. The particle diameter of nanoparticles was calculated from the XRD pattern, according to the Scherrer equation, and the samples were calculated by the particle size ranges of the nanoparticles at 6 nm, 6 nm, 8 nm, and 10.3 nm. The calculated sizes coincided with the TEM results. The above results indicated that the NPs had a uniform morphology and the average sizes of the shells had not changed.

### 3.2. Optical Properties

#### 3.2.1. Effect of Ce^3+^ Concentration on the Luminescence Properties of BaLuF_5_:Yb^3+^,Er^3+^ NPs

**Figure 3:**
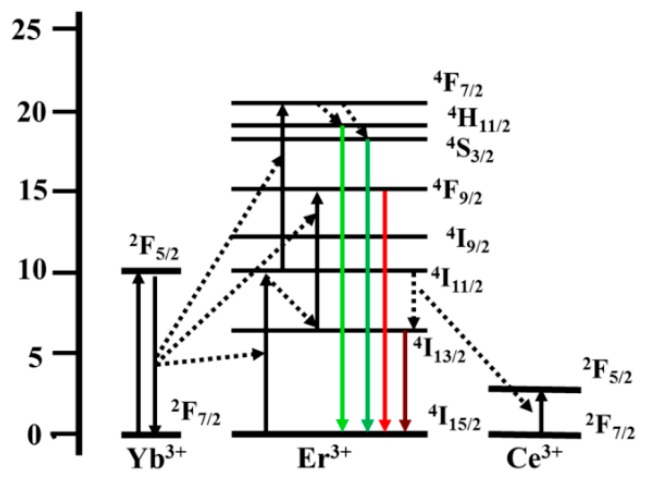
Diagram of energy levels of Yb^3+^-Er^3+^-Ce^3+^ and up-conversion (UC) emission and down-conversion (DC) emission processes in the BaLuF_5_:Yb^3+^,Er^3+^,Ce^3+^ systems under 980 nm excitation.

In BaLuF_5_:Yb^3+^,Er^3+^,Ce^3+^ systems, all the energy transfer processes are shown in the Figure 3. With the excitation of a 980 nm laser diode, the Yb^3+^ ions are excited from the ^2^F_7/2_ level to the ^2^F_5/2_ level and then transfer the energy to Er^3+^ to populate higher energy levels of the Er^3+^ ions:^4^H_11/2_ level, ^4^F_9/2_, and ^4^F_7/2_. The ^4^H_11/2_ → ^4^I_15/2_ (≈525 nm), ^4^S_3/2_ → ^4^I_15/2_ (≈545 nm), and ^4^F_9/2_ → ^4^I_15/2_ (≈655 nm) transitions gives the UC emission, and the ^4^I_13/2_ → ^4^I_15/2_ transition gives the DC emission at 1.53 μm. Interestingly, with the addition of Ce^3+^ ions, the energy transfer occurs between Ce^3+^ and Er^3+^:^4^I_11/2_ (Er^3+^) + ^2^F_5/2_ (Ce^3+^) → ^4^I_13/2_ (Er^3+^) + ^2^F_7/2_ (Ce^3+^). Furthermore, the ^4^I_11/2_ state of Er^3+^ ions populate the ^4^I_13/2_ state [17,35,36,37]. The intensity of the DC emission is enhanced and that of the UC emission are suppressed by the addition of Ce^3+^ ions. 

**Figure 4:**
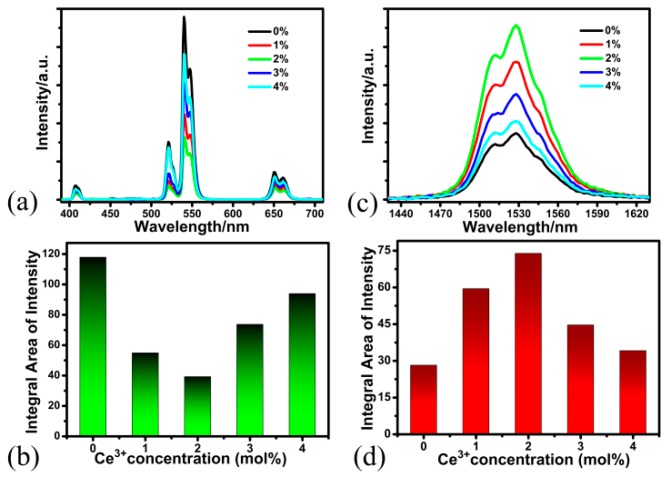
(**a**) UC and (**c**) DC emission spectra of BaLuF_5_:18%Yb^3+^,2%Er^3+^,*x*% Ce^3+^ NPs with different Ce^3+^ concentrations (*x* = 0, 1, 2, 3, and 4) under the excitation of a 980 nm laser diode. Intensity enhancement of (**b**) UC and (**d**) DC emission depending on the Ce^3+^ concentrations in BaLuF_5_:18%Yb^3+^,2%Er^3+^,*x*%Ce^3+^ NPs.

In order to investigate the effect of Ce^3+^ ion on the UC emission, we measured the UC emission spectra of BaLuF_5_:18%Yb^3+^,2%Er^3+^,*x*%Ce^3+^ NPs with different Ce^3+^ concentrations (*x* = 0, 1, 2, 3, and 4) under the excitation of a 980 nm laser diode, and the data is shown in Figure 4a. All samples exhibit several UC emission peaks, which are attributed to the ^4^H_11/2_ → ^4^I_15/2_ (≈525 nm), ^4^S_3/2_ → ^4^I_15/2_ (≈545 nm), and ^4^F_9/2_ → ^4^I_15/2_ (≈655 nm) transitions of Er^3+^ ions, respectively. When the Ce^3+^ concentration was 0%, the UC luminescence of BaLuF_5_:Yb^3+^,Er^3+^ NPs was the strongest one. It is clear that the intensity of the UC emissions decreased gradually with the increase of Ce^3+^ concentration from 0% to 2% (as shown in Figure 4b). This is due to the following energy transfer occurring between Ce^3+^ and Er^3+^:^4^I_11/2_ (Er^3+^) + ^2^F_5/2_ (Ce^3+^) → ^4^I_13/2_ (Er^3+^) + ^2^F_7/2_ (Ce^3+^) [17,35,36,37]. However, when the concentration of Ce^3+^ ions reached 4%, the intensity of the UC emissions increased monotonically (as shown in Figure 4b). The above results show that doping Ce^3+^ ions led to the suppression of the UC emissions of Er^3+^ ions.

In addition, we also studied the influence of the Ce^3+^ concentration on the DC luminescence of BaLuF_5_:Yb^3+^,Er^3+^ NPs. BaLuF_5_:18%Yb^3+^,2%Er^3+^,*x*%Ce^3+^ (*x* = 0, 1, 2, 3, and 4) NPs were synthesized using a high-boiling solvent method. Figure 4c shows the DC emission of the ^4^I_13/2_ → ^4^I_15/2_ transition of Er^3+^ ions with varying Ce^3+^ concentration under the excitation of a 980 nm laser. We found that the intensity of the DC luminescence gradually increased with increasing Ce^3+^ concentration from 0% to 2% (as shown in Figure 4d). This may be due to the branching ratio of the Er^3+^:^4^I_11/2_ → ^4^I_13/2_ transition, which can be increased by doping with Ce^3+^ ions, and the energy transfer process can increase the population of ^4^I_13/2_ state of Er^3+^ ions through the following energy transfer process: ^4^I_11/2_ (Er^3+^) + ^2^F_5/2_ (Ce^3+^) → ^4^I_13/2_ (Er^3+^) + ^2^F_7/2_ (Ce^3+^) [17,35,36,37]. The results led to the enhancement of the DC emission of Er^3+^ ions. Meanwhile, the intensity of the DC emissions reduced monotonically with increasing Ce^3+^ concentration from 2% to 4% (as shown in Figure 4d), since the cross relaxation: Er^3+^:^4^I_13/2_ + Ce^3+^: ^2^F_5/2_ → Er^3+^:^4^I_15/2_ + Ce^3+^: ^2^F_7/2_ happened. These results indicate that when the concentration of Ce^3+^ ions was 2%, the intensity of the DC luminescence reached its maximum. The DC emissions of BaLuF_5_:18%Yb^3+^,2%Er^3+^,2%Ce^3+^ NPs were about 2.6 times compared to that of BaLuF_5_:18%Yb^3+^,2%Er^3+^ NPs, which means that doping Ce^3+^ ions led to the enhancement of the DC emissions of Er^3+^ ions. Thus, the optimum concentration of Er^3+^ was about 2% for tri-doped BaLuF_5_ NPs.

#### 3.2.2. Effect of Yb^3+^ Concentration of the Shell on the DC Luminescence Properties of BaLuF_5_:Yb^3+^,Er^3+^,Ce^3+^@BaLuF_5_:Yb^3+^ Core-Shell NPs

**Figure 5:**
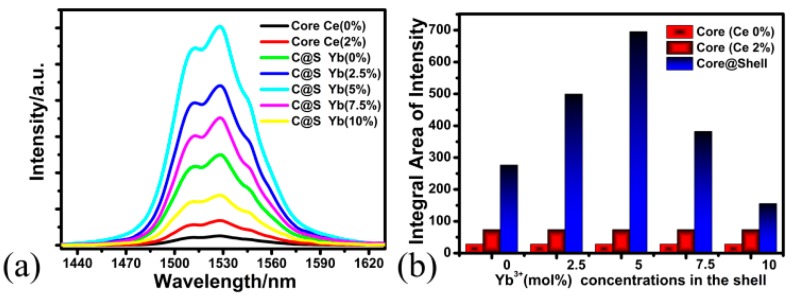
(**a**) DC emission spectra of BaLuF_5_:18%Yb^3+^,2%Er^3+^ core NPs, BaLuF_5_:18%Yb^3+^,2%Er^3+^,2%Ce^3+^ core NPs and BaLuF_5_:18%Yb^3+^,2%Er^3+^,2%Ce^3+^@BaLuF_5_:*x*%Yb^3+^ NPs (*x* = 0, 2.5, 5, 7.5 and 10) core-active-shell NPs under the excitation of a 980 nm laser diode. (**b**) Intensity enhancement of DC emission depending on the Yb^3+^ concentrations in BaLuF_5_ core-active-shell NPs.

Here, we choose BaLuF_5_:18%Yb^3+^,2%Er^3+^,2%Ce^3+^ NPs as the core, and prepared BaLuF_5_:18%Yb^3+^,2%Er^3+^,2%Ce^3+^@BaLuF_5_:*x*%Yb^3+^ (*x* = 0, 2.5, 5, 7.5 and 10) core-shell NPs. To clarify the effects of Yb^3+^ concentration on the shell on the DC luminescence properties of BaLuF_5_:18%Yb^3+^,2%Er^3+^,2%Ce^3+^@BaLuF_5_:Yb^3+^ core-shell NPs, we measured the DC emission spectra of the core-shell NPs with different Yb^3+^ concentrations (0%, 2.5%, 5%, 7.5%, and 10%) under a 980 nm laser excitation, and the measured data is shown in Figure 5a. We can see from the Figure 5a that BaLuF_5_:Yb^3+^,Er^3+^ core NPs and BaLuF_5_:Yb^3+^,Er^3+^,Ce^3+^ core NPs show the weakest emission peak at 1530 nm. The main reason is that the surface area-to-volume ratio of the core-only NPs was very high and a large portion of the dopants should be located at the surface. Hence, the energy from the pump light will be easily quenched by the surface defects of the core-only NPs. The luminescence intensity of BaLuF_5_ core-inert-shell NPs was obviously increased after the BaLuF_5_ insert shell. The luminescence intensity of BaLuF_5_ core-inert-shell NPs was increased by 3.7 times compared to that of the BaLuF_5_:Yb^3+^,Er^3+^ core NPs with doping Ce^3+^ ions. This is because the insert shell can suppress the nonradiative transitions [23,24]. Interestingly, when the shell was doped with Yb^3+^ ions, the luminescence intensity of the BaLuF_5_:Yb^3+^,Er^3+^,Ce^3+^ core-active-shell NPs could be increased further compared to that of the BaLuF_5_:Yb^3+^,Er^3+^,Ce^3+^ core-insert-shell NPs. The luminescence intensity of the BaLuF_5_:Yb^3+^,Er^3+^,Ce^3+^ core-active-shell NPs monotonically enhanced with increasing of Yb^3+^ concentration in the shell from 0% to 5% (as shown in Figure 5b). However, when the Yb^3+^ concentration in the shell was 5%, the luminescence intensity of the BaLuF_5_ core-shell NPs reached its maximum value. This was due to the Yb^3+^ ions in the shell could transfer energy from the pump source to the core and make a contribution to the DC emissions [31]. When the Yb^3+^ concentration in the shell continuously increased from 5% to 10%, the luminescence intensity of the BaLuF_5_:Yb^3+^,Er^3+^,Ce^3+^ core-active-shell NPs gradually decreased since the concentration quenching effect occurred [31,38]. These results indicate that the optimum concentration of Yb^3+^ in the shell was about 5% for BaLuF_5_:Yb^3+^,Er^3+^,Ce^3+^ core-active-shell NPs, the intensity of the UC emissions of BaLuF_5_:Yb^3+^,Er^3+^,Ce^3+^@BaLuF_5_:Yb^3+^ core-active-shell NPs was increased by 9.4 times compared that of the BaLuF_5_:Yb^3+^,Er^3+^,Ce^3+^ core NPs, and was increased by 24.6 times compared to that of the BaLuF_5_:Yb^3+^,Er^3+^ core NPs without doping Ce^3+^ ions.

#### 3.2.3. Synthesis of BaLuF_5_:Yb^3+^,Er^3+^,Ce^3+^@BaLuF_5_:Yb^3+^@BaLuF_5_:Yb^3+^ Core-Shell-Shell NPs with Strong Down-Conversion Luminescence

**Figure 6:**
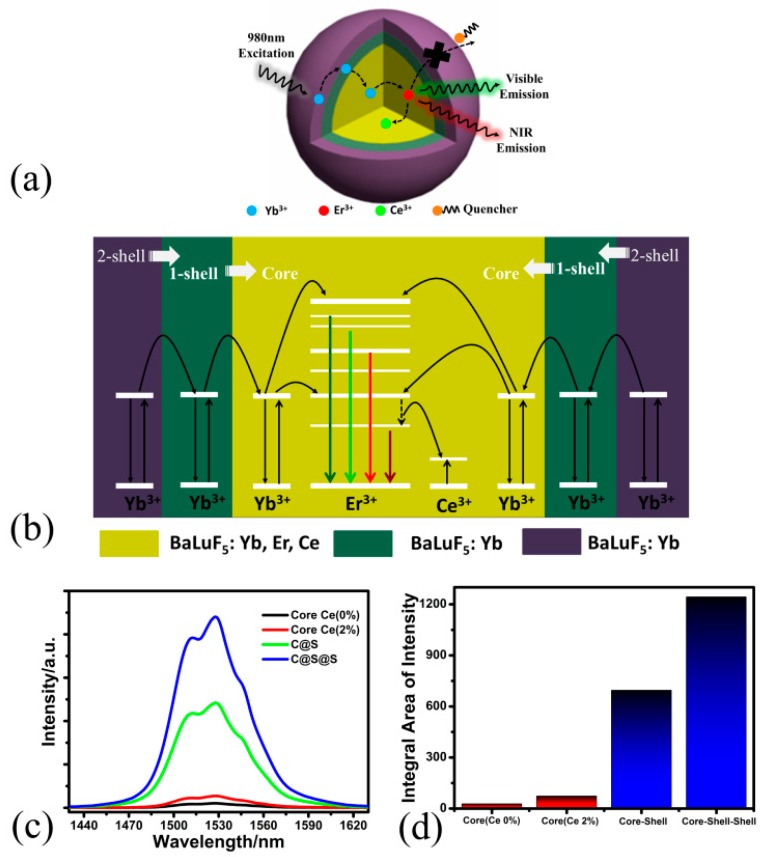
(**a**) Schematic illustration and (**b**) energy transfer mechanisms of BaLuF_5_:18%Yb^3+^,2%Er^3+^,2%Ce^3+^@BaLuF_5_:5%Yb^3+^@BaLuF_5_:5%Yb^3+^ core-active-shell-active-shell NPs. (**c**) DC emission spectra of BaLuF_5_:18%Yb^3+^,2%Er^3+^ core NPs, BaLuF_5_:18%Yb^3+^,2%Er^3+^2%Ce^3+^ core NPs, BaLuF_5_:18%Yb^3+^,2%Er^3+^,2%Ce^3+^@BaLuF_5_:5%Yb^3+^ core-active-shell NPs and BaLuF_5_:18%Yb^3+^,2%Er^3+^,2%Ce^3+^@BaLuF_5_:5%Yb^3+^@BaLuF_5_:5%Yb^3+^ core-active-shell-active-shell NPs. (**d**) Intensity enhancement of DC emission in all the above NPs.

In order to get the tri-doped BaLuF_5_:Yb^3+^,Er^3+^,Ce^3+^ core-shell NPs with strong DC luminescence, we synthesized BaLuF_5_:18%Yb^3+^,2%Er^3+^,2%Ce^3+^@BaLuF_5_:5%Yb^3+^@BaLuF_5_:5%Yb^3+^ core-shell-shell NPs via a high boiling solvent process through a layer-by-layer strategy. Figure 6c shows the DC emission of BaLuF_5_:18%Yb^3+^,2%Er^3+^ core NPs, BaLuF_5_:18%Yb^3+^,2%Er^3+^,2%Ce^3+^ core NPs, BaLuF_5_:18%Yb^3+^,2%Er^3+^,2%Ce^3+^@BaLuF_5_:5%Yb^3+^ core-active-shell NPs and BaLuF_5_:18%Yb^3+^,2%Er^3+^,2%Ce^3+^@BaLuF_5_:5%Yb^3+^@BaLuF_5_:5%Yb^3+^ core-active-shell-active-shell NPs under the excitation of a 980 nm laser diode. The DC emission intensity of BaLuF_5_ core NPs without doping Ce^3+^ ions shows the weakest DC emission. By doping with Ce^3+^ ions, the luminescence intensity of the BaLuF_5_:Yb^3+^,Er^3+^,Ce^3+^ core NPs was 2.6 times more than that of BaLuF_5_:Yb^3+^,Er^3+^ core NPs due to the energy transfer between Er^3+^ and Ce^3+^. After, by growing an BaLuF_5_:5%Yb^3+^active shell, the DC emission intensity of the BaLuF_5_:Yb^3+^,Er^3+^,Ce^3+^ core-active-shell NPs was further enhanced, since the active shell could be used to not only suppress surface quenching but also transfer energy from the pump light to the core region efficiently through Yb^3+^ ions inside the active shells. When the number of the active shell layers reaches two, the DC emission intensity of the BaLuF_5_:Yb^3+^,Er^3+^,Ce^3+^ core-active-shell-active-shell NPs was 16.8 times more than that of BaLuF_5_:Yb^3+^,Er^3+^,Ce^3+^ core NPs. This was because the size of the active shell was too small to completely suppress surface quenching, and coating two active shell layers could effectively suppress surface quenching [31]. In the last, we got BaLuF_5_:18%Yb^3+^,2%Er^3+^,2%Ce^3+^@BaLuF_5_:5%Yb^3+^@BaLuF_5_:5%Yb^3+^ core-active-shell-active-shell NPs with the strong DC luminescence at 1.5 μm. The DC emission intensity of the BaLuF_5_:18%Yb^3+^,2%Er^3+^,2%Ce^3+^@BaLuF_5_:5%Yb^3+^@BaLuF_5_:5%Yb^3+^ core-active-shell-active-shell NPs was 44 times more than that of BaLuF_5_:Yb^3+^,Er^3+^ core NPs without the dopant Ce^3+^ ions. Besides, we measured the photo stability of the BaLuF_5_:18%Yb^3+^,2%Er^3+^,2%Ce^3+^@BaLuF_5_:5%Yb^3+^@BaLuF_5_:5%Yb^3+^ core-active-shell-active-shell NPs (as shown in Figure S1). Those results the show that the core-active-shell-active-shell NPs have optical stability.

**Figure 7:**
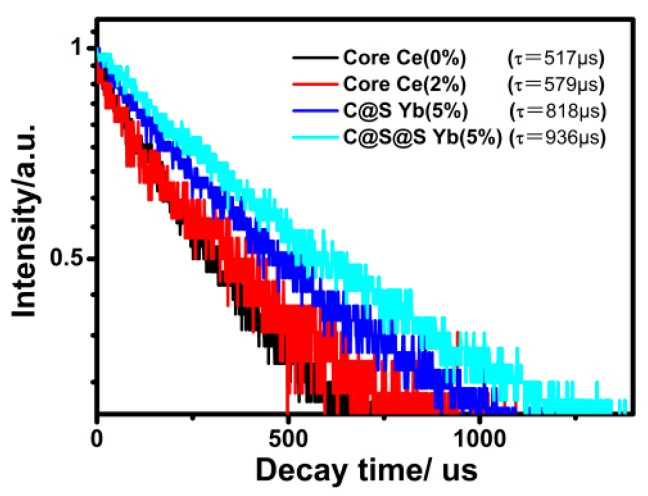
The lifetime of the ^4^I_13/2_ level of Er^3+^ (monitored at 1530 nm corresponding to the ^4^I_13/2_ → ^4^I_13/2_) in BaLuF_5_:18%Yb^3+^,2%Er^3+^ NPs, BaLuF_5_:18%Yb^3+^,2%Er^3+^,2%Ce^3+^ NPs, BaLuF_5_:18%Yb^3+^,2%Er^3+^,2%Ce^3+^@BaLuF_5_:5%Yb^3+^ NPs, and BaLuF_5_:18%Yb^3+^,2%Er^3+^,2%Ce^3+^@BaLuF_5_:5%Yb^3+^@BaLuF_5_:5%Yb^3+^ NPs by using a 980 nm pulsed laser with a pulse width of 100 μs and a repetition rate of 20 Hz as the excitation source.

In addition, we measured the lifetime of the ^4^I_13/2_ level of Er^3+^ in BaLuF_5_:18%Yb^3+^,2%Er^3+^ NPs, BaLuF_5_:18%Yb^3+^,2%Er^3+^,2%Ce^3+^ NPs, BaLuF_5_:18%Yb^3+^,2%Er^3+^,2%Ce^3+^@BaLuF_5_:5%Yb^3+^ NPs, and BaLuF_5_:18%Yb^3+^,2%Er^3+^,2%Ce^3+^@BaLuF_5_:5%Yb^3+^@BaLuF_5_:5%Yb^3+^ NPs by using a 980 nm pulsed laser with a pulse width of 100 μs and a repetition rate of 20 Hz as the excitation source. The result is shown in Figure 7. Each of the decay curves could be fitted well with a single-exponential function as *I* = *I*_0_ exp(*−t*/*τ*), where *I_0_* is the initial emission intensity at *t* *=* 0 and *τ* is the lifetime of the monitored level. Obviously, the lifetime of the ^4^I_13/2_ level of Er^3+^ was extended from 517 μs to 579 μs by introducing Ce^3+^ ions into the BaLuF_5_: Yb^3+^,Er^3+^ core NPs. This was because the quenching of Er^3+^ ions from the ^4^I_11/2_ state to the ^4^I_13/2_ state by the energy transfer occurs between Ce^3+^ and Er^3+^: ^4^I_11/2_ (Er^3+^) + ^2^F_5/2_ (Ce^3+^) → ^4^I_13/2_ (Er^3+^) + ^2^F_7/2_ (Ce^3+^). Interestingly, by growing a BaLuF_5_: 5% Yb^3+^ shell on the core NP, the lifetime of the ^4^I_13/2_ level was extended from 579 μs to 818 μs owing to the reduction of the nonradiative relaxation rate caused by the surface passivation. When the number of the shell layer reached two, the lifetime of the ^4^I_13/2_ level further increased to 936 μs since the thickness of each shell layer was about 2 nm, and therefore one shell layer was not enough for suppressing surface quenching completely. The result agreed well with the tendency toward the dependence of the measured DC emissions (shown in Figure 6c).

## 4. Conclusions

In summary, we synthesized BaLuF_5_:18%Yb^3+^,2%Er^3+^,2%Ce^3+^ core NPs by introducing Ce^3+^ ions via a high boiling solvent process. In the case of BaLuF_5_:18%Yb^3+^,2%Er^3+^ core NPs, the UC emission intensity of BaLuF_5_:18%Yb^3+^,2%Er^3+^,2%Ce^3+^ core NPs significantly decreased and the DC emission intensity obviously increased due to the energy transfer between Er^3+^ and Ce^3+^ ions according to: ^4^I_11/2_ (Er^3+^) + ^2^F_5/2_ (Ce^3+^) → ^4^I_13/2_ (Er^3+^) + ^2^F_7/2_ (Ce^3+^). We prepared BaLuF_5_:Yb^3+^,Er^3+^,Ce^3+^ core-active-shell-active-shell NPs via a layer-by-layer strategy. In comparison with the optical properties of BaLuF_5_:Yb^3+^,Er^3+^ core NPs, the DC emission intensity of BaLuF_5_:Yb^3+^,Er^3+^,Ce^3+^ core-active-shell-active-shell NPs were enhanced by 44 times after coating with two-layer BaLuF_5_:Yb^3+^ active shells. We effectively enhanced the DC emission intensity of Yb^3+^-Er^3+^ co-doping BaLuF_5_ NPs through introducing Ce^3+^ ions into BaLuF_5_ NPs and multiple BaLuF_5_:5%Yb^3+^ active-shell coatings.

## Supplementary Materials

The following are available online at http://www.mdpi.com/2079-4991/8/8/615/s1, Figure S1: (a) The intensity change of BaLuF_5_:18%Yb^3+^,2%Er^3+^,2%Ce^3+^@BaLuF_5_:5%Yb^3+^@BaLuF_5_:5%Yb^3+^ NPs core-active-shell-active-shell NPs for 980 nm constant light exposure. (b) The inset shows the DC emission of the core-active-shell-active-shell NPs after 980 nm laser light irradiation for 0 h, 6 h, 12 h, respectively.
