# Supplementary material for: Synthesis of Small Ce3+-Er3+-Yb3+ Tri-Doped BaLuF5 Active-Core-Active-Shell-Active-Shell Nanoparticles with Strong Down Conversion Luminescence at 1.5 μm
Source: Nanomaterials (Basel). 2018 Aug 14;8(8):615. doi: 10.3390/nano8080615 (PMC6116297; doi:10.3390/nano8080615)
Supplement: Supplementary file 1 [file nanomaterials-08-00615-s001.pdf]

nanomaterials

Supplementary Materials: Synthesis of Small Ce^{3+}-Er^{3+}-Yb^{3+} Tri-Doped $BaLuF_5$ Active-core-active-shell-active-shell Nanoparticles with Strong Down Conversion Luminescence at 1.5 μm

Yongling Zhang, Yudi Shi, Zhengkun Qin, Mingxing Song and Weiping Qin

We measured the photo stability of $BaLuF_5$:18%Yb^{3+},2%Er^{3+},2%Ce^{3+}@$BaLuF_5$:5%Yb^{3+}@$BaLuF_5$:5% Yb^{3+} core-active-shell-active-shell NPs. Figure S1a shows the intensity change of $BaLuF_5$:18%Yb^{3+},2% Er^{3+},2%Ce^{3+}@$BaLuF_5$:5%Yb^{3+}@$BaLuF_5$:5%Yb^{3+} core-active-shell-active-shell NPs for 980 nm constant light exposure. The inset shows the down conversion (DC) emission of the core-active-shell-active-shell NPs after 980 nm laser light irradiation for 0 h, 6 h, and 12 h, respectively (Figure S1b). The intensity of the core-active-shell-active-shell NPs has no change. That results the show $BaLuF_5$:18%Yb^{3+},2%Er^{3+},2%Ce^{3+}@$BaLuF_5$:5%Yb^{3+}@$BaLuF_5$:5%Yb^{3+} core-active-shell-active-shell NPs have optical stability.

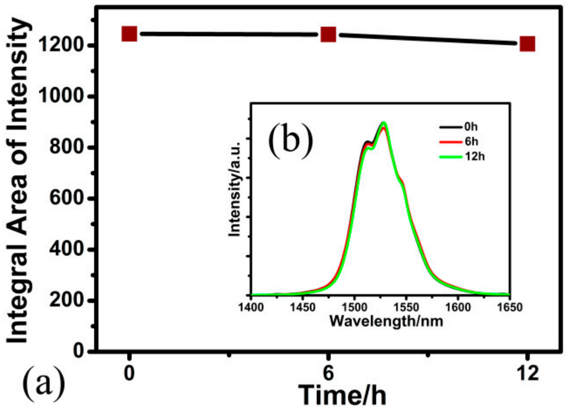

Figure S1. (**a**) The intensity change of $BaLuF_5$:18%Yb^{3+},2%Er^{3+},2%Ce^{3+}@$BaLuF_5$:5%Yb^{3+}@$BaLuF_5$:5%Yb^{3+} core-active-shell-active-shell NPs for 980 nm constant light exposure. (**b**) The inset shows the down conversion (DC) emission of the core-active-shell-active-shell NPs after 980 nm laser light irradiation for 0 h, 6 h, and 12 h, respectively.
